# Range-Wide Nuclear Phylogeography of the Aesculapian Snake (*Zamenis longissimus*) and the Evolutionary Significance of a Northern Relict River-Valley Population System

**DOI:** 10.3390/genes17101265

**Published:** 2026-10-12

**Authors:** Petr Kotlík, Radka Musilová, David Fischer, Silvia Marková, Ivan Rehák

**Affiliations:** 1 Laboratory of Molecular Ecology, Institute of Animal Physiology and Genetics, Czech Academy of Sciences, 277 21 Liběchov, Czech Republic; markova@iapg.cas.cz; 2 Faculty of Environmental Sciences, Czech University of Life Sciences Prague, 165 00 Praha-Suchdol, Czech Republic; musilovaradka@fzp.czu.cz; 3 Mining Museum Příbram, 261 01 Příbram, Czech Republic; fischer-david@seznam.cz; 4 Prague Zoo, 171 00 Prague, Czech Republic; ivan.rehak@zoopraha.cz

**Keywords:** snakes, Ophidia, phylogeography, *SPTBN1*, nucleotide polymorphisms, contact zone, conservation genetics, conservation management, Holocene climatic optimum, climate change

## Abstract

Background/Objectives: Relict populations can preserve genetic legacies that illuminate species history and inform conservation. Isolated northern populations of the Aesculapian snake (*Zamenis longissimus*) are considered remnants of a broader Holocene distribution, offering an opportunity to explore historical population connections and their conservation significance. We assessed diversity in Czech populations and provide the first range-wide phylogeographic perspective based on the nuclear marker *SPTBN1*. Methods: We analyzed mitochondrial DNA (mtDNA) and *SPTBN1* in Czech populations, including the newly confirmed Běšický chochol locality (BCH), and assembled a range-wide *SPTBN1* dataset. Results: Across Europe, *SPTBN1* haplotypes s1 and s2 showed an east–west phylogeographic pattern that only partly corresponded to the major mitochondrial lineages; a previously unreported haplotype (s4) was identified in western Slovakia. In Bohemia (western Czech Republic), both SPTBN1 haplotypes co-occurred with Eastern and Western mitochondrial lineages at two lower Ohře River localities, Želinský meander (ZM) and BCH, whereas the upstream Stráž nad Ohří and introduced Central Bohemian populations contained only the Eastern mitochondrial lineage and nuclear haplotype s1. The Moravian population (eastern Czech Republic) carried both nuclear haplotypes but only Eastern-lineage mtDNA, demonstrating that nuclear haplotype coexistence alone is not diagnostic of contact between mitochondrial lineages. Conclusions: The genetic legacy of historical lineage contact extends beyond ZM to BCH. Together with the historical landscape configuration of the Ohře River valley, these findings support the interpretation that both localities are surviving fragments of a more extensive Holocene population system. Their retention of multiple components of evolutionary diversity highlights the conservation value of these northern relicts and warrants explicit consideration in future management.

## 1. Introduction

Conservation biology increasingly recognizes that preserving biodiversity requires more than preventing species extinctions. Genetic diversity provides the raw material for adaptation, while phylogeographic structure preserves a legacy of the historical demographic and climatic processes that have shaped present-day biodiversity. Populations that retain unique evolutionary lineages or genetic signatures of historical demographic processes may therefore represent important priorities for conservation management [1,2].

Many contemporary populations occur in landscapes shaped by both natural environmental change and human activities. Climatic fluctuations have repeatedly driven range expansions, contractions, and secondary contact between previously isolated lineages, while more recent habitat loss and fragmentation have reduced formerly continuous populations to small, isolated remnants [3,4]. These processes have left complex spatial patterns of genetic diversity that can provide insights into both historical demographic processes and their consequences for contemporary populations.

Relict and peripheral populations are particularly informative in this context. Although often small and vulnerable, they may preserve distinctive genetic variation and evolutionary legacies that are poorly represented elsewhere [5,6]. Populations within or near historical contact zones may be especially informative because they can retain genetic signatures of interactions among lineages with distinct evolutionary histories [7,8]. Understanding and conserving such populations can therefore contribute both to reconstructing past demographic processes and to preserving the evolutionary diversity generated by them.

The Aesculapian snake (*Zamenis longissimus*) provides an excellent model for investigating the evolutionary and conservation significance of relict populations. The species is a thermophilous snake inhabiting structurally diverse habitats, including woodland edges, scrub and semi-open landscapes [9]. It is one of Europe’s largest snakes, reaching approximately 2 m in total length, and feeds primarily on small mammals and birds. Mitochondrial DNA (mtDNA) analyses have identified four major phylogeographic lineages corresponding to separate Pleistocene refugia, where populations persisted during glacial periods and from which postglacial range expansion subsequently occurred [9]. The Western and Eastern lineages are widespread across Europe west and south, and east of the Alps, respectively, whereas the Greek and Asian lineages have more restricted distributions. Although the species remains relatively widespread across southern and south-eastern Europe, populations near the northern range margin are highly fragmented and often isolated from the continuous distribution [10,11]. Many of these northern populations are regarded as relicts of a formerly broader Holocene distribution [11,12]. Their present occurrence reflects the combined effects of postglacial expansion, subsequent climatic deterioration and more recent anthropogenic habitat modification. Consequently, they may preserve genetic signatures of historical demographic processes that are poorly represented elsewhere within the species’ range [13].

The Aesculapian snake is strictly protected under Annex IV of the EU Habitats Directive, and a European action plan has been developed for its conservation [10]. At the northern margin of its range in the Czech Republic, the species is legally protected and classified as Critically Endangered, and a national conservation action plan coordinated by the Nature Conservation Agency of the Czech Republic was established in 2008 [14,15]. The recent recognition of substantial phylogeographic differentiation among these northern populations adds an evolutionary dimension to their established conservation importance [13].

**Figure 1:**
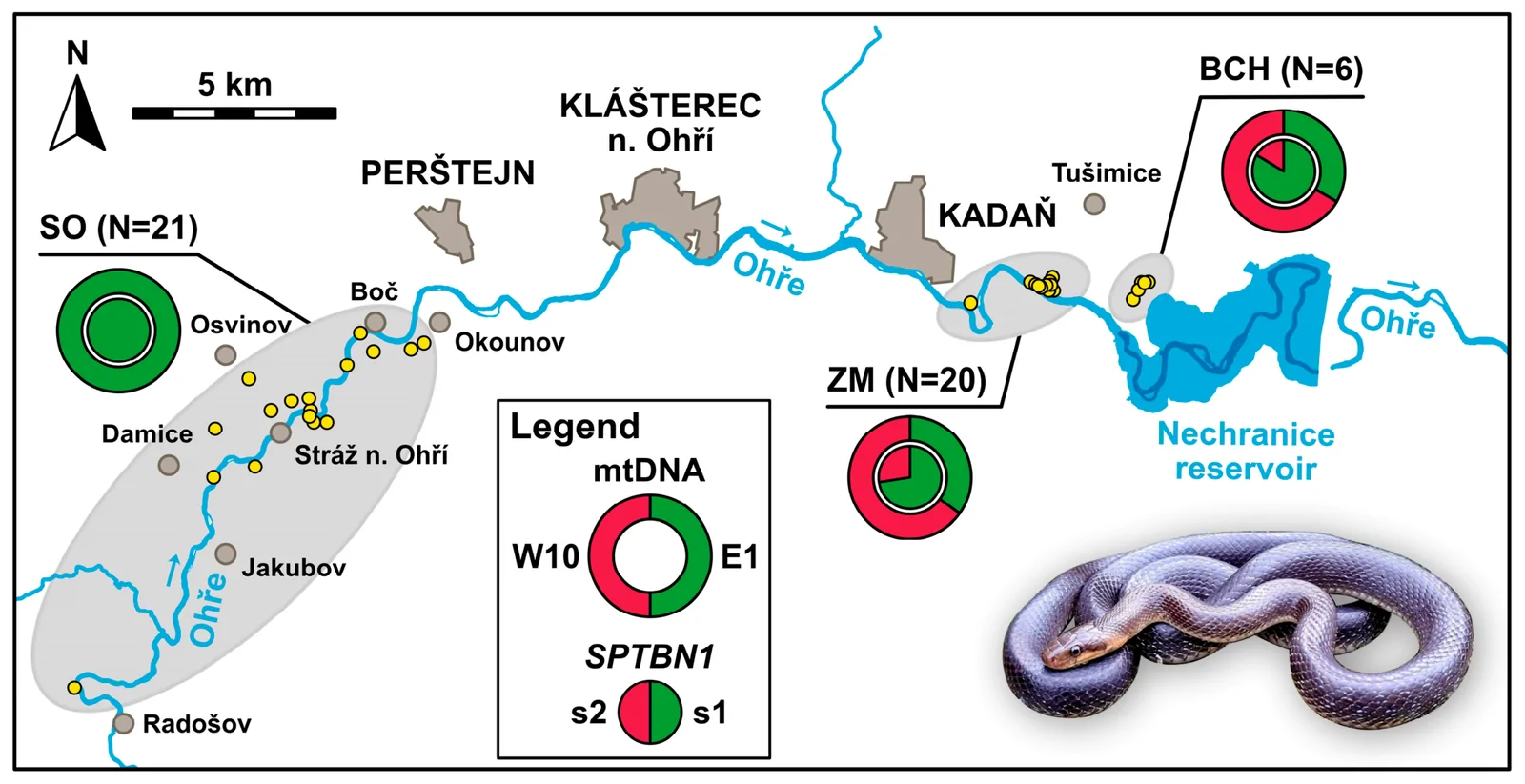
Location and genetic composition of *Zamenis longissimus* populations along the Ohře River in Bohemia (western Czech Republic). Pie charts show mtDNA (outer rings) and *SPTBN1* (inner rings) haplotype frequencies at Stráž nad Ohří (SO), Želinský meander (ZM), and Běšický chochol (BCH); sample sizes are given in parentheses. Yellow circles indicate sampling sites, and gray ellipses schematically indicate the approximate extent of the populations based on known sampling localities. The pre-dam course of the Ohře River is shown within the Nechranice Reservoir. The inset photograph shows a *Z. longissimus* individual from SO.

Within the Czech Republic, populations in Moravia (eastern Czech Republic) remain connected to the continuous range extending through Slovakia and Austria, whereas populations in Bohemia (western Czech Republic) are geographically isolated [16,17]. Two natural populations occur along the Ohře River in northwestern Bohemia: Stráž nad Ohří (SO) in the middle Ohře River valley and Želinský meander (ZM), situated approximately 20 km downstream, in the lower Ohře valley (Figure 1). A third Bohemian population in Central Bohemia (CB), in the Vltava River basin and surrounding landscapes, is considered to have originated through human-mediated introduction, sourced from SO, in the early 1980s [13].

The Ohře populations are of particular interest because they represent northern relicts occupying a river corridor that has undergone major environmental transformation. ZM is located approximately 4 km upstream of the Nechranice Reservoir, constructed between 1961 and 1968, which inundated a formerly meandering section of the lower Ohře valley. Reconstructions of the pre-reservoir landscape [18,19] indicate that the area now occupied by the reservoir formed an extensive meandering river valley with geomorphological characteristics similar to those preserved at ZM today (Figure 1). Its inundation substantially transformed the river corridor and contributed to the recent fragmentation of a formerly more extensive network of suitable riverine and thermophilous habitats.

**Figure 2:**
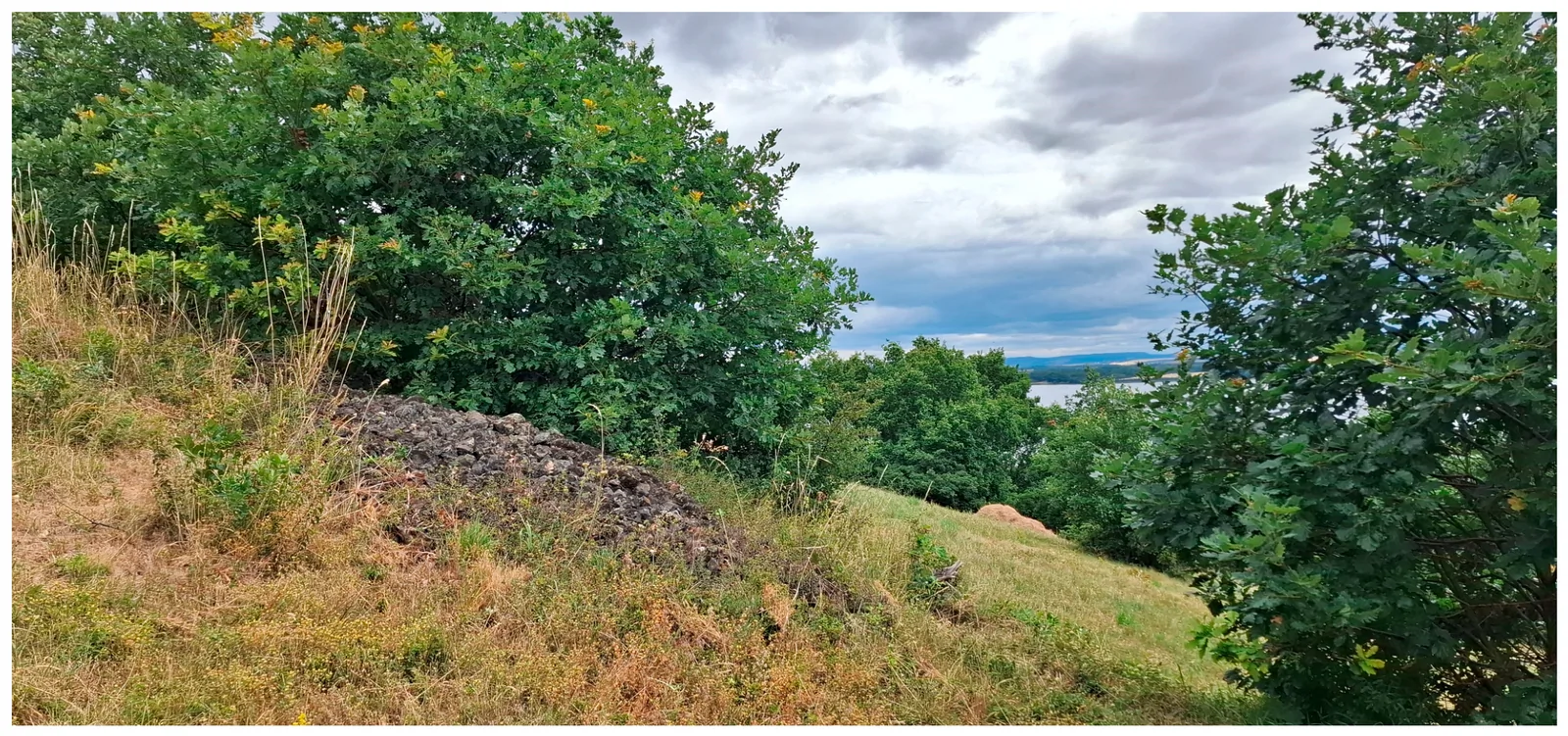
Běšický chochol locality (BCH), with the Nechranice Reservoir visible in the background. Photograph taken on 7 July 2025.

**Figure 3:**
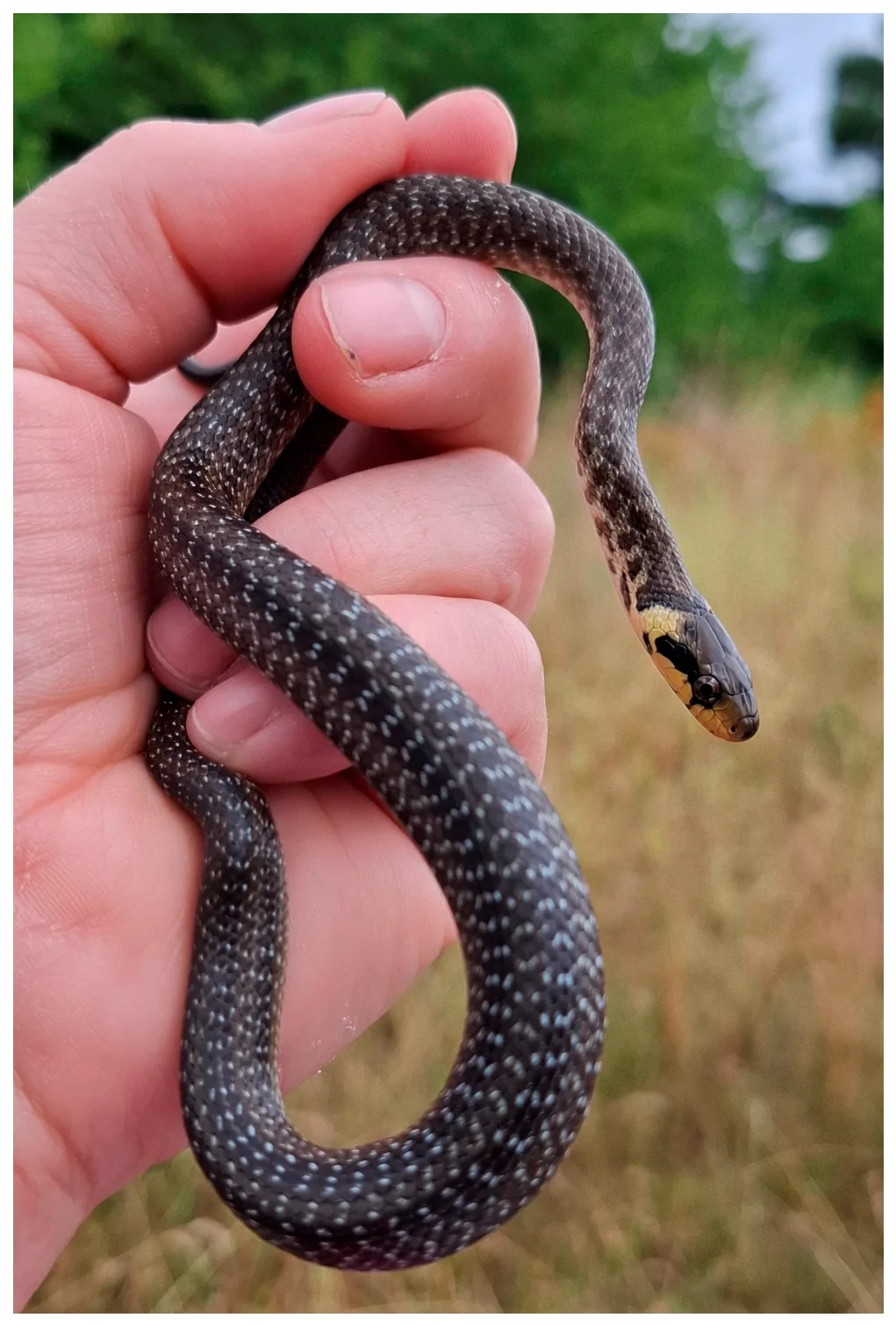
A juvenile *Zamenis longissimus* from Běšický chochol (BCH), photographed on 7 July 2025.

Recent field surveys aimed at verifying the current distribution of the species in the region ([20]; authors’ observations) confirmed the occurrence of *Z. longissimus* at an additional locality, Běšický chochol (BCH) (Figure 2); an individual from this locality is shown in Figure 3. The locality lies on a peninsula extending into the Nechranice Reservoir (Figure 1). Because most reports of Aesculapian snakes outside known populations remain unconfirmed, verification of the species at a new locality is of considerable conservation interest. Its position within the inundated former river corridor raises the possibility that it represents another remnant of a historically more extensive population system in the Ohře valley. The locality therefore provides an opportunity to test this hypothesis genetically.

**Figure 4:**
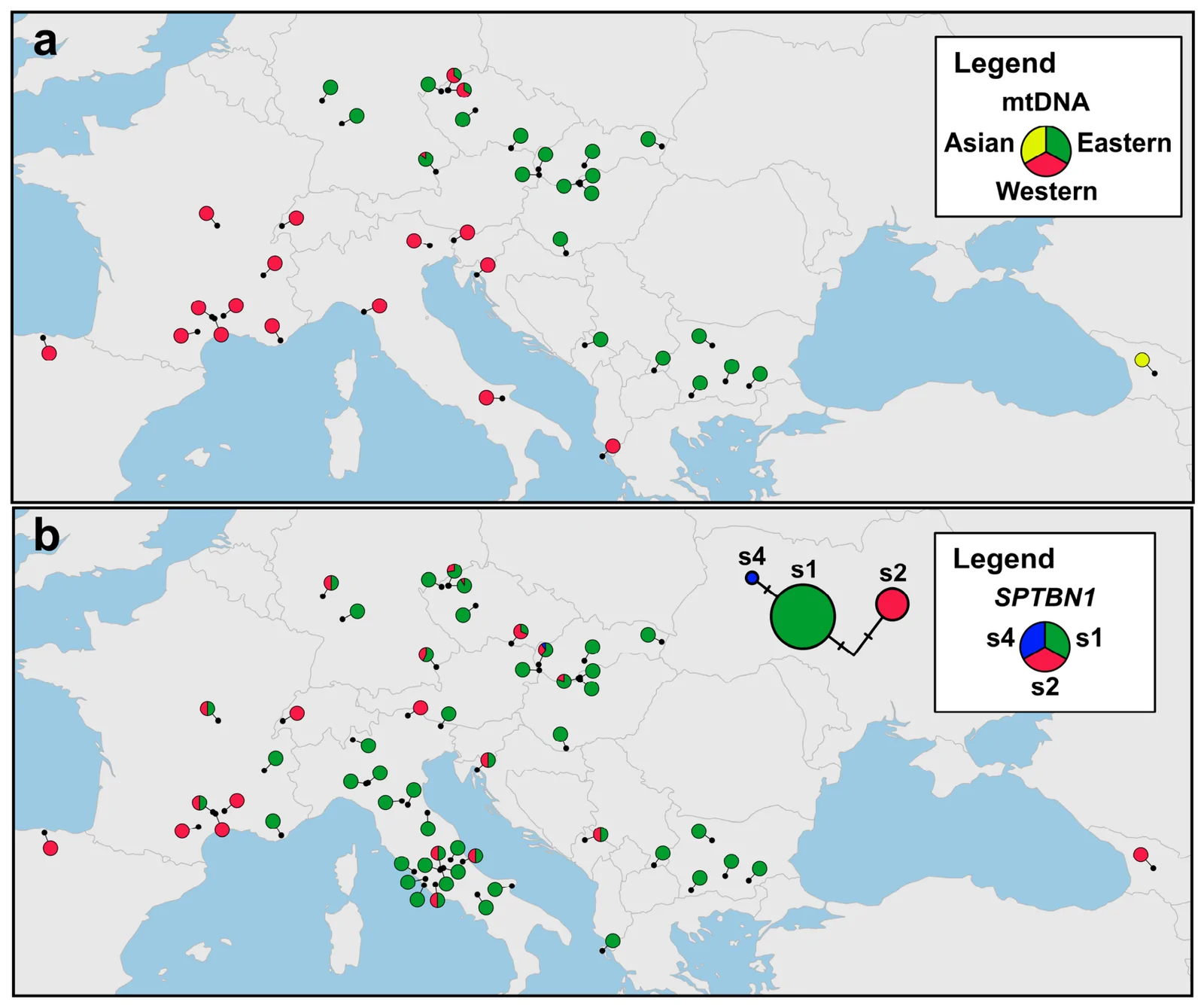
Geographic distribution of (**a**) mitochondrial DNA (mtDNA) and (**b**) *SPTBN1* haplotypes of *Zamenis longissimus* across the populations included in the nuclear dataset. The same samples are represented in both panels, except for Italy, where mtDNA and *SPTBN1* data originate from different sampling localities reported in [9] and [24], respectively. The pie charts on the maps are schematic in size and are not proportional to sample sizes; frequencies represent individuals for mtDNA and allele copies for *SPTBN1*. The inset in (**b**) shows a tree of the three *SPTBN1* haplotypes; circle sizes in the tree denote relative haplotype frequencies, crossbars indicate nucleotide substitutions, and the break indicates the root.

Recent mtDNA analyses [13] revealed an exceptional phylogeographic pattern within the ZM population: individuals belonging to both the Eastern and Western phylogeographic lineages occur at this locality (Figure 1), representing the first confirmed record of the Western lineage in the Czech Republic and only the second known population across the species’ range in which these two major mitochondrial clades coexist [9] (Figure 4a). Their coexistence was interpreted as a legacy of historical secondary contact during the broader Holocene distribution of the species in Central Europe [13]. In contrast, the upstream SO population contains only the Eastern lineage despite its close geographic proximity [13].

However, mtDNA represents only a single maternally inherited locus. Nuclear loci generally exhibit weaker phylogeographic structure because of their larger effective population size and slower lineage sorting, while biparental inheritance allows them to reflect gene flow through both sexes [21,22]. Comparisons of mitochondrial and nuclear variation can therefore provide complementary insights into historical isolation, retention of ancestral polymorphism, and subsequent population contact [23]. Two haplotypes of the nuclear gene *spectrin beta, non-erythrocytic 1* (*SPTBN1*), have previously been described in Italian populations of *Z. longissimus* [24], but their distribution across the remainder of the species’ range is unknown. It therefore remains unclear whether *SPTBN1* variation retains a signature of the major refugial divisions identified by mtDNA or reflects a different evolutionary history.

Here, we analyze variation at *SPTBN1* in all Bohemian populations previously examined using mtDNA, include the newly confirmed BCH locality and the Moravian population from Podyjí National Park connected to the continuous range, and assemble a range-wide *SPTBN1* dataset spanning the European distribution of *Z. longissimus*. This enables the first species-wide comparison of mitochondrial phylogeographic structure and nuclear genetic variation in the Aesculapian snake. We ask whether the exceptional mitochondrial diversity of the Ohře River populations is accompanied by elevated nuclear diversity, how *SPTBN1* variation is geographically structured across Europe and relates to the major mitochondrial lineages, and what the combined evidence reveals about the evolutionary history and conservation significance of the Bohemian relict populations.

## 2. Materials and Methods

### 2.1. Study Populations and Sampling

The present study includes new samples from three natural Bohemian localities: Stráž nad Ohří (SO), Želinský meander (ZM) and Běšický chochol (BCH), together with samples previously analyzed for mtDNA by Rehák et al. [13] from SO, ZM and the introduced Central Bohemian population (CB). The new material comprised 11 individuals from SO, nine from ZM and six from BCH, sampled during 2025–2026. *SPTBN1* was analyzed in 61 Bohemian individuals (SO: 21; ZM: 20; BCH: 6; CB: 14) across both sampling sets. Paired mtDNA and *SPTBN1* data were available for all 61 Bohemian individuals. Two additional CB individuals had previously published mtDNA data only.

Sampling, tissue collection, DNA extraction, and mitochondrial lineage assignment followed the protocols described by Rehák et al. [13]. DNA was obtained from non-destructively collected samples, comprising ventral scale clips (approximately 2 mm^2^) and cotton-tipped oral swabs (one swab per individual), and from tail-tip tissue samples (approximately 5 mm long) collected from road-killed juveniles. All samples were preserved in ethanol prior to laboratory analyses. Genomic DNA was extracted using the DNeasy Blood & Tissue Kit (Qiagen, Hilden, Germany), following the manufacturer’s protocol, as described by Rehák et al. [13]. All procedures were carried out in accordance with ethical standards to minimize animal stress and in compliance with Czech legislation (permit numbers: KK/1098/ZZ/20-4, KUUK/057806/2021, and 099683/2024/KUSK). The samples used in this study were obtained during routine monitoring activities conducted as part of the national biodiversity monitoring program coordinated by the Nature Conservation Agency of the Czech Republic.

### 2.2. Mitochondrial DNA Analysis

To assess mtDNA variation in newly collected samples and determine phylogeographic lineage affiliation, fragments of the *cytochrome c oxidase subunit I* (*COI*) and *cytochrome b* (*cytb*) genes were amplified and sequenced. For *COI*, a 522 bp fragment was amplified using newly designed primers 5F20 (5′-TGGTACAGGATGAACCGTCT-3′) and 506R21 (5′-CCTACCGTGAATATGTGGTGA-3′). These primers were designed to improve amplification reliability compared with those previously used [9]. For *cytb*, amplification was performed using primers L14910 and H16064, following the protocol previously described for *Z. longissimus* by Musilová et al. [9] and subsequently used by Rehák et al. [13]. The annealing temperature was 55 °C for *COI* and 45 °C for *cytb*. Sequencing of the PCR products was performed at Macrogen Europe (Amsterdam, The Netherlands) on an ABI 3730xl System (Applied Biosystems, Foster City, CA, USA).

### 2.3. Nuclear DNA Analysis

Variation in two nuclear markers, *SPTBN1* and *c-mos*, was examined. Amplification and sequencing of both loci followed the protocols and primers reported by Salvi et al. [24]. The annealing temperature was 55 °C for both *SPTBN1* and *c-mos*. Because no sequence variation was detected in *c-mos* among the analyzed *Z. longissimus* samples, subsequent analyses focused on *SPTBN1*. To assess geographic variation in *SPTBN1* across the species’ range, we analyzed 73 archived DNA extracts from the study of Musilová et al. [9]. The archived material included eight Czech samples, all from Podyjí (Moravia). Samples representing the Greek mitochondrial lineage could not be analyzed because insufficient DNA was available.

PCR products were purified and sequenced in both directions. Chromatograms were inspected and assembled in CodonCode Aligner v. 11.0.1 (CodonCode Corporation, Centerville, MA, USA). Heterozygous sites were detected as double peaks using the “Find Mutations” function in CodonCode Aligner and confirmed by visual inspection of the chromatograms. The PCR product from one heterozygous individual from BCH was cloned using the QIAGEN PCR Cloning Plus Kit (Qiagen, Hilden, Germany), following the manufacturer’s protocol, and sequenced, confirming the presence of haplotypes s1 and s2. Individuals showing heterozygosity at the same two sites were therefore assigned the s1/s2 genotype.

### 2.4. Data Analysis

For mitochondrial analyses, the *COI* and *cytb* sequences obtained from each individual were aligned with the sequences reported by Rehák et al. [13]. The alignment comprised 481 bp of COI and 1102 bp of cytb. The newly generated sequences were assigned to previously identified mitochondrial haplotypes based on sequence identity, and phylogeographic lineage nomenclature followed Musilová et al. [9] and Rehák et al. [13]. Mitochondrial haplotype and lineage frequencies were summarized separately for each population. For ZM, the lineage frequencies obtained from the newly collected samples were compared with those reported from the previous sampling by Rehák et al. [13] to assess whether the coexistence of the Eastern and Western lineages was consistently recovered. The mitochondrial composition of BCH was subsequently compared with that of the previously characterized Bohemian populations.

The final *SPTBN1* alignment comprised 846 bp. The sequences were compared with the s1 and s2 haplotypes reported by Salvi et al. [24], and haplotypes were assigned according to the two diagnostic nucleotide positions distinguishing them [24]. Because s3 was previously assigned to *Z. lineatus* [24], the novel *Z. longissimus* haplotype identified in this study was designated s4. Haplotype s4 differed from s1 by a single substitution from G to A at position 726. To assess the range-wide geographic structure of *SPTBN1* variation, haplotype counts and frequencies were summarized by sampling locality based on allele counts (two alleles per individual) and compared with the mitochondrial lineage assignments previously established for the same populations [9,13].

Conformance of *SPTBN1* genotype frequencies to Hardy–Weinberg equilibrium was assessed using a two-sided conditional exact test with standard exact *p*-values. Associations between mitochondrial lineage and *SPTBN1* genotype were evaluated separately within ZM and BCH. At ZM, a Fisher–Freeman–Halton exact test was applied to a 2 × 3 contingency table comprising the two mitochondrial lineages and the three *SPTBN1* genotypes (s1/s1, s1/s2 and s2/s2). At BCH, where only s1/s1 and s1/s2 genotypes were observed, the association was evaluated using a two-sided Fisher’s exact test. Statistical significance was assessed at α = 0.05.

Relationships among *SPTBN1* haplotypes were visualized as a haplotype tree based on nucleotide differences among haplotypes [25].

## 3. Results

### 3.1. Mitochondrial Phylogeographic Variation

Analysis of newly collected samples corroborated the mitochondrial phylogeographic pattern previously reported by Rehák et al. [13]. The upstream SO population and the introduced CB population were represented exclusively by the Eastern lineage (haplotype E1), whereas Eastern (E1) and Western (W10) mitochondrial lineages co-occurred at both ZM and the newly documented BCH locality (Figure 1).

Among the newly analyzed ZM samples, three individuals carried haplotype E1 and six carried haplotype W10, compared with four E1 and seven W10 individuals in the previous sampling. Similarly, BCH comprised two individuals with haplotype E1 and four with haplotype W10.

### 3.2. Range-Wide Distribution of SPTBN1 Haplotypes

To place the Bohemian populations into a broader phylogeographic context, we generated *SPTBN1* sequences for samples representing the European distribution of *Z. longissimus* previously characterized using mtDNA by Musilová et al. [9], together with the published Italian sequences of Salvi et al. [24]. Across the combined dataset, three *SPTBN1* haplotypes (s1, s2, and s4) were identified (Figure 4b). The haplotypes s1 and s2 corresponded to the haplotypes reported for *Z. longissimus* by Salvi et al. [24] and were defined by two SNPs. A third, previously unreported haplotype (s4) was identified in a single s1/s4 heterozygote from the Little Carpathians in western Slovakia, as indicated by a clear double peak at a single nucleotide position in both forward and reverse sequences.

The geographic distribution of *SPTBN1* showed a broad east–west pattern partly resembling the mitochondrial phylogeography (Figure 4a), but the distributions of the common haplotypes s1 and s2 did not strictly correspond to the mitochondrial lineages (Figure 4b). The s1 haplotype predominated in the core of the Eastern mitochondrial lineage, including Bulgaria and the Carpathian populations of Slovakia. In western Europe, s2 was frequent or exclusive in the sampled populations from France, Spain and Switzerland. However, most Italian populations belonging to the Western mitochondrial lineage were dominated by s1, while populations from Central Europe, including Germany, Hungary and southern Moravia, contained both s1 and s2. The Caucasian population belonging to the Asian mitochondrial lineage carried only s2.

### 3.3. Nuclear Variation in Czech Populations

Within the broader European context, the Czech populations showed contrasting distributions of the s1 and s2 haplotypes (Figure 1 and Figure 4b). The SO and CB populations were fixed for the s1 haplotype, whereas both s1 and s2 were detected in ZM and BCH.

The ZM population comprised ten s1/s1 homozygotes, nine s1/s2 heterozygotes and one s2/s2 homozygote. No significant deviation from Hardy–Weinberg equilibrium was detected (two-sided exact test, *p* = 1.000), although statistical power was limited by the small sample size. No significant association was detected between mitochondrial lineage and *SPTBN1* genotype (Fisher–Freeman–Halton exact test, *p* = 0.446). Both Eastern and Western mitochondrial lineages occurred among s1/s1 homozygotes and s1/s2 heterozygotes, whereas the single s2/s2 homozygote carried the Western mitochondrial lineage.

At BCH, five individuals were s1/s1 homozygotes and one was an s1/s2 heterozygote; no s2/s2 homozygotes were detected. Genotype frequencies did not deviate from Hardy–Weinberg equilibrium (two-sided exact test, *p* = 1.000), although statistical power was limited by the small sample size. The single heterozygote carried the Eastern mitochondrial lineage, while the five s1/s1 homozygotes comprised one Eastern- and four Western-lineage individuals. No significant association was detected between mitochondrial lineage and *SPTBN1* genotype (two-sided Fisher’s exact test, *p* = 0.333).

The Moravian population from Podyjí National Park also contained both *SPTBN1* haplotypes s1 and s2 despite being represented exclusively by the Eastern mitochondrial lineage [9], consistent with the broader pattern observed in Central Europe (Figure 4b). Thus, the lower Ohře River localities ZM and BCH were the only Czech populations in which both Eastern and Western mitochondrial lineages co-occurred with both *SPTBN1* haplotypes.

## 4. Discussion

### 4.1. Range-Wide Nuclear Pattern Is Related to but Distinct from mtDNA Phylogeography

Our study provides the first range-wide perspective on the geographic structure of nuclear genetic variation in *Z. longissimus*. Although *SPTBN1* exhibited a broad east–west geographic pattern partly paralleling mtDNA phylogeography, the nuclear haplotypes s1 and s2 were not strictly partitioned among the mitochondrial lineages. Whereas mtDNA is divided into geographically coherent Western, Eastern and Asian clades, *SPTBN1* haplotypes s1 and s2 occur across multiple refugial regions. The predominance of s1 in both the Italian and Balkan refugial areas, together with the higher frequency of s2 in western and Central Europe and its occurrence in the Caucasus region in Georgia, may reflect the persistence of ancestral nuclear polymorphism and/or subsequent gene flow.

Mitonuclear discordance is expected because mitochondrial and nuclear loci differ in their modes of inheritance, effective population sizes, and coalescent histories [26,27]. The larger effective population size of autosomal loci results in slower lineage sorting, allowing ancestral nuclear polymorphisms to persist across population divergences [28], while historical gene flow can further decouple nuclear genealogies from mitochondrial phylogeographic structure [23].

### 4.2. Czech Populations in a European Mitonuclear Phylogeographic Context

Among the isolated Bohemian populations, mitochondrial and nuclear markers show a remarkably similar spatial pattern (Figure 1 and Figure 4). Both mtDNA lineages and *SPTBN1* haplotypes s1 and s2 occur in the lower Ohře River populations (ZM and BCH), whereas the upstream SO population and the introduced CB population are uniform for both markers. However, the range-wide comparison shows that s1 and s2 are not restricted to populations where divergent mitochondrial lineages coexist. The Moravian population in Podyjí, despite belonging exclusively to the Eastern mitochondrial lineage, contains both s1 and s2, as do several other Central European populations (Figure 4). The co-occurrence of s1 and s2 therefore cannot by itself be attributed to contact between the major mitochondrial lineages. Rather, the nuclear data indicate that the lower Ohře populations have retained elevated nuclear diversity alongside their exceptional mitochondrial phylogeographic composition [13].

### 4.3. Genetic Legacy of the Historical Ohře River Population System

Several independent lines of evidence support the interpretation that the present-day lower Ohře populations are remnants of a historically more extensive population system. Genetically, the coexistence of Eastern and Western mitochondrial lineages originally reported from ZM [13] was independently confirmed in newly collected material at similar frequencies. The same mitochondrial composition, together with both s1 and s2 *SPTBN1* haplotypes, was detected at BCH, demonstrating that this exceptional genetic pattern is not restricted to a single locality but characterizes a broader section of the lower Ohře River valley.

The geomorphological history of the valley provides an independent line of support. Before construction of the Nechranice Reservoir (1961–1968), ZM and BCH formed part of an extensive meandering river corridor with geomorphological characteristics resembling those preserved at ZM today [18,19]. Reservoir construction inundated much of this landscape (Figure 1) and, together with subsequent industrial development, contributed to the fragmentation of suitable riverine habitats. ZM and BCH are therefore more plausibly interpreted as surviving fragments of this former river-valley population system than as independent populations of recent origin.

Historical occurrence records further support this scenario. Records dating to the late nineteenth century, summarized by Rehák [29] and Zavadil et al. [14,17,30], document *Z. longissimus* throughout the middle Ohře region, from the vicinity of Karlovy Vary downstream towards Kadaň and including sites now inundated by the Nechranice Reservoir. Together with the recent documentation of BCH, these records indicate that the species formerly occupied a substantially larger section of the Ohře River valley than represented by the surviving populations today.

This historically broader distribution also included the upstream SO population, which today lacks both the Western mitochondrial lineage and the s2 nuclear haplotype. The contrasting genetic composition of SO and the lower Ohře populations suggests that different parts of the historical Ohře population system underwent different demographic histories following fragmentation. This may reflect founder effects, genetic drift or long-term restriction of gene flow along the river corridor, although distinguishing among these alternatives will require denser sampling across the historical range.

Taken together, the genetic, geomorphological and historical evidence supports the interpretation that ZM and BCH represent surviving fragments of a formerly more extensive Holocene population system, whose origins may extend to the broader Holocene distribution of the species. Within this system, the coexistence of the two deeply divergent mitochondrial lineages is most parsimoniously interpreted as a legacy of historical contact between lineages originating from different glacial refugia, whereas the *SPTBN1* data demonstrate the retention of elevated nuclear variation without uniquely identifying the demographic process responsible for it. The lower Ohře populations therefore preserve multiple components of the evolutionary diversity generated during the species’ postglacial history.

### 4.4. Conservation Implications

Our results highlight the importance of relict populations as reservoirs of evolutionary diversity shaped by historical demographic processes. Although small, geographically isolated populations are often emphasized for their vulnerability [2], they may also preserve unique combinations of genetic variation that have disappeared from larger, more continuous populations [5,6].

The lower Ohře River populations of *Z. longissimus* exemplify this principle by preserving both the exceptional coexistence of Eastern and Western mitochondrial lineages and elevated nuclear diversity absent from the other isolated Bohemian populations. 

From a conservation perspective, maintaining both the genetic diversity and demographic viability of the Ohře populations should be a priority. Earlier accounts and monitoring indicate declining abundance in the SO population [15,29], while Musilová et al. [16] suggested that the geographic isolation of the Poohří population may have resulted in reduced genetic variability through inbreeding and a population bottleneck. Translocation of Aesculapian snakes from South Moravia to the Poohří region has consequently been considered as a means of addressing declining abundance and potential inbreeding. However, the discovery of substantial genetic diversity and its geographic structuring among the Ohře populations calls for careful reassessment of such translocations. Any translocation should be preceded by an explicit evaluation of demographic need, the genetic compatibility of source and recipient populations, and the potential benefits and risks of genetic rescue [31,32]. Because the genetically diverse ZM and BCH populations were recognized only recently, their long-term demographic trajectories remain largely unknown. Future monitoring should therefore encompass the entire Ohře population system. Management should prioritize maintaining population sizes and connectivity among the surviving Ohře localities, while ensuring that any proposed introduction does not inadvertently erode their structured genetic diversity.

More generally, our study demonstrates the value of integrating mitochondrial phylogeography, nuclear genetic variation, historical occurrence records and landscape reconstruction when assessing the conservation significance of relict populations. Our findings also underscore the value of sustained collaboration among evolutionary biologists, field zoologists and conservation practitioners: linking genetic research with long-term monitoring and management can uncover previously unrecognized components of biodiversity and provide a stronger scientific basis for conservation decisions.

## 5. Conclusions

Our range-wide analysis of *SPTBN1* variation in *Z. longissimus* revealed a geographic pattern only partly corresponding to the major mitochondrial phylogeographic lineages, indicating that nuclear and mitochondrial variation retain different aspects of the species’ evolutionary history. In the lower Ohře River valley, the coexistence of Eastern and Western mitochondrial lineages together with elevated nuclear diversity supports the interpretation that these populations are surviving fragments of a formerly more extensive river-valley population system. These populations preserve multiple components of the species’ postglacial evolutionary diversity that should be considered in future monitoring and conservation management.

## Data Availability

Sequences generated in this study have been deposited in GenBank (accession numbers QB078485–QB078487).

## Abbreviations

The following abbreviations are used in this manuscript:

BCH	Běšický chochol
CB	Middle Povltaví and lower Posázaví region in Central Bohemia
mtDNA	Mitochondrial DNA
SO	Municipality of Stráž nad Ohří and its surroundings
ZM	Želinský meander
